# Evolution of small prokaryotic genomes

**DOI:** 10.3389/fmicb.2014.00742

**Published:** 2015-01-06

**Authors:** David J. Martínez-Cano, Mariana Reyes-Prieto, Esperanza Martínez-Romero, Laila P. Partida-Martínez, Amparo Latorre, Andrés Moya, Luis Delaye

**Affiliations:** ^1^Departamento de Ingeniería Genética, Cinvestav Unidad IrapuatoIrapuato, Mexico; ^2^Institut Cavanilles de Biodiversitat i Biologia Evolutiva, Universitat de ValenciaValencia, Spain; ^3^Centro de Ciencias Genómicas, Universidad Nacional Autónoma de MéxicoCuernavaca, Mexico

**Keywords:** reductive genome evolution, endosymbiosis, minimal genome size, streamlining evolution, Black Queen Hypothesis, Muller’s ratchet, robustness-based selective reduction, symbionelle

## Abstract

As revealed by genome sequencing, the biology of prokaryotes with reduced genomes is strikingly diverse. These include free-living prokaryotes with ∼800 genes as well as endosymbiotic bacteria with as few as ∼140 genes. Comparative genomics is revealing the evolutionary mechanisms that led to these small genomes. In the case of free-living prokaryotes, natural selection directly favored genome reduction, while in the case of endosymbiotic prokaryotes neutral processes played a more prominent role. However, new experimental data suggest that selective processes may be at operation as well for endosymbiotic prokaryotes at least during the first stages of genome reduction. Endosymbiotic prokaryotes have evolved diverse strategies for living with reduced gene sets inside a host-defined medium. These include utilization of host-encoded functions (some of them coded by genes acquired by gene transfer from the endosymbiont and/or other bacteria); metabolic complementation between co-symbionts; and forming consortiums with other bacteria within the host. Recent genome sequencing projects of intracellular mutualistic bacteria showed that previously believed universal evolutionary trends like reduced G+C content and conservation of genome synteny are not always present in highly reduced genomes. Finally, the simplified molecular machinery of some of these organisms with small genomes may be used to aid in the design of artificial minimal cells. Here we review recent genomic discoveries of the biology of prokaryotes endowed with small gene sets and discuss the evolutionary mechanisms that have been proposed to explain their peculiar nature.

## INTRODUCTION

Darwin proposed an externalist theory of evolution where organisms provide the raw material and the environment selects (Gould, 2002). The outcome of this process is a fine adjustment of organisms to the environment. The evolution of prokaryotes with reduced genomes is not an exception to this Darwinian principle. Host-associated bacteria and archaea evolved the smallest genomes in nature other than those of organelles and viruses. The rationale of this pattern is simple. Prokaryotes living in a protected and chemically rich medium can afford losing more genes than those coping with the vagaries of a free-living lifestyle (Morowitz, 1993). On the other hand, different lineages of free-living bacteria, most of them in marine environments, evolved reduced genomes likely by the direct action of natural selection (Giovannoni et al., 2014).

## WHAT IS THE MINIMAL GENOME SIZE FOR EXTANT FREE-LIVING PROKARYOTES?

Previous surveys indicated that free-living prokaryotes had no less than ∼1,300 genes (Islas et al., 2004; Podar et al., 2008; Delaye et al., 2010). However, recent metagenomic sequencing suggests that there are free-living Actinobacteria with approximately 800 genes. This was discovered at the Mediterranean Sea and the bacteria were named “*Candidatus* Actinomarina minuta” (Ghai et al., 2013). Surprisingly, it is also one of the smallest cells with a cell volume of only ∼0.013 μm^3^. If further sequencing of its complete genome confirms this estimate (and it is very likely that it will do), it will sensibly change our knowledge about the minimum number of genes a cell needs to survive in present free-living conditions, in a similar fashion than the discovery of “*Candidatus* Carsonella ruddii” shook our belief of the minimal gene set required for cells in 2006 (Nakabachi et al., 2006; McCutcheon and Moran, 2012). Meanwhile, as reviewed below, there exists a diversity of lineages of free-living prokaryotes that converged to approximately 1,300 genes despite their varying phylogenetic origins and nutritional strategies.

Nowadays, *Methanothermus fervidus* with a genome coding for 1,311 proteins and 50 RNA genes, stands as the free-living archaeon (that does not grow associated to another cell) with the smallest sequenced genome. This organism is a methanogen and was isolated from an anaerobic Icelandic spring (Anderson et al., 2010). As mentioned above, other groups of free-living prokaryotes evolved similar genome sizes, several of them from marine environments.

For instance, α-proteobacteria from clade SAR11, which is the most abundant group of heterotrophic bacteria in the oceans, are endowed with genomes ranging from 1,321 to 1,541 protein-coding genes (Grote et al., 2012). Among them, is “*Candidatus* Pelagibacter ubique” HTCC1062, which is one of the most studied members of clade SAR11 and is an oligotroph with 1,354 protein-coding genes that generates energy by respiration (Giovannoni et al., 2005).

Another group of marine prokaryotes that evolved similar genome sizes are the β-proteobacteria from clade OM43. Specifically strains HTCC2181 and HIMB624 that have 1,377 and 1,381 protein-coding genes respectively (Giovannoni et al., 2008; Huggett et al., 2012). These are marine and freshwater bacteria that live heterotrophically by using methylated compounds as carbon sources (Giovannoni et al., 2008; Huggett et al., 2012).

Contrasting with the previously mentioned heterotrophs whose smallest genomes have ∼1,300 genes, photoautotrophic free-living bacteria have larger genomes. For instance, some strains from *Prochlorococcus marinus*, the most abundant photosynthetic organism on Earth, have genomes coding for as few as 1,716 protein-coding genes (Dufresne et al., 2003; Rocap et al., 2003; Scanlan et al., 2009).

And finally, non-marine bacteria with small genomes include the mollicute *Acholeplasma laidlawii*, which is a saprophyte and opportunistic parasite found in a wide variety of environments and has a genome coding for 1,380 protein-coding genes (Windsor et al., 2010; Lazarev et al., 2011); the lactobacilli *Weissella koreensis* KACC 15510 which is a heterotroph that participates in the fermentation of kimchi (a representative Korean fermented food) and has a genome coding for 1,335 predicted protein-coding sequences (Lee et al., 2011); the dehalorespiration *Dehalococcoides* sp. BAV1 with a genome coding for 1,371 protein-coding genes and a member of the Chloroflexi (He et al., 2003; Löﬄer et al., 2013); and the Chrenarchaeon *Desulfurococcus mucosus* O7/1 with its ability for sulfur respiration with a genome with 1,371 protein-coding genes (Wirth et al., 2011).

Why do different lineages of free-living cultivable bacteria have genomes with no less than 1,300 genes? One possible explanation is that extant biotic and abiotic environments exert a selective pressure against simpler cells, therefore imposing an ecological limit on the minimum complexity necessary for a cell to survive (Gould, 1996). The idea is that free-living cells with fewer genes are outcompeted by cells with a more complete genetic arsenal, unless associated to other organisms. However, intuitive this idea is, still requires experimental validation.

However, it is important to take into consideration that it is possible that our sample of genome sequences from cultivable organisms does not accurately represents the distribution of genome sizes that exist on nature (Giovannoni et al., 2014). Additionally, as suggested by metagenomic data, there may exist a whole biodiversity of uncultivable bacteria with genomes with less than 1,300 genes, as seems to be the case of “*Ca*. Actinomarina minuta” (Ghai et al., 2013).

In this direction, a note of caution regarding the limit of ∼1,300 genes for free-living prokaryotes is given by *Lactobacillus fermentum* CECT 5716, a hetero-fermentative lactic acid bacterium inhabiting human mucosal surfaces and breast milk. This bacterium has a genome with 1,109 protein-coding genes (Jiménez et al., 2010). And, although this organism would be classified as a symbiont because it is naturally associated to humans, it grows well under laboratory conditions (Jiménez, personal communication) thus blurring the distinction between free-living and host-associated microorganisms. The discovery of “*Ca*. Actinomarina minuta,” as well as the existence of *L*. *fermentum* CECT 5716, indicates that in the near future we will probably discover free-living bacteria with smaller genomes.

## DRIVERS OF GENOME REDUCTION AMONG FREE-LIVING PROKARYOTES

**FIGURE 1:**
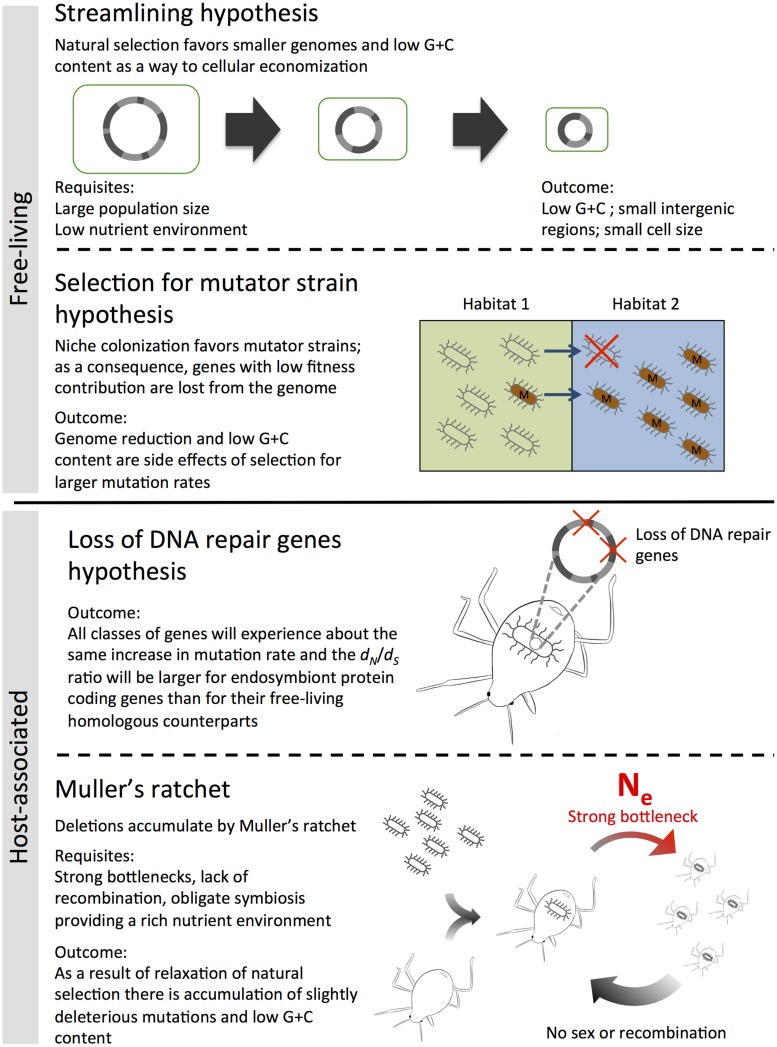
**Streamlining and Muller’s ratchet hypotheses are commonly used to explain genome reduction in free-living and host-associated bacteria respectively.** Alternative hypotheses include “selection for mutator strains” and “loss of DNA repair genes.”

As we will describe below, different mechanisms have been proposed to account for genome reduction among free-living prokaryotes (**Figure 1**; Mira et al., 2001; Dufresne et al., 2005; Giovannoni et al., 2005; Marais et al., 2008; Morris et al., 2012).

### THE STREAMLINING HYPOTHESIS

Genome reduction by a process known as streamlining, in which smaller genomes are favored directly by selection as a way to cellular economization, is perhaps the most popular explanation (Mira et al., 2001; Dufresne et al., 2005). According to this hypothesis, natural selection directly favors genome reduction in free-living prokaryotes living in low-nutrient environments (Mira et al., 2001; Grote et al., 2012). This claim is based on the basic idea that superfluous genes are eliminated because they confer a fitness cost to the bacterium. This is especially effective in large population sizes. The reasoning is as follows: very large population sizes render negligible the effect of genetic drift and more importantly, render highly efficient the process of natural selection. Then, when a fitness-increasing deletion occurs it is quickly fixed in the population, especially under high selective pressures such as low-nutrient environments where small genomes evolve as a way to economize on matter and energy for cell maintenance (Dufresne et al., 2005; Koskiniemi et al., 2012).

For instance, streamlining was suggested to be responsible of genome reduction in the high-light adapted marine cyanobacteria *P. marinus* MED4 and the low-light adapted *P. marinus* SS120 (Dufresne et al., 2003, 2005). According to the streamlining hypothesis the following characteristics are consistent with natural selection acting to economize cellular metabolism. First, the small G+C content of these genomes (∼30 to 36%) contributes to fewer requirements for phosphorus and nitrogen for DNA synthesis which are scarce in the environment where *P. marinus* MED4 lives (Dufresne et al., 2005). Second, the small cell volume of *P. marinus* SS120 (∼0.1 μm^3^) is suggested to improve photosynthetic efficiency by reducing self-shading and enhancing nutrient uptake by increasing the surface-to-volume ratio for the cell, and also it is in itself an adaptation that has in turn exerted an evolutionary pressure for a smaller genome (Dufresne et al., 2003). This follows the logic that a smaller bacterial cell has a smaller volume and can only contain a small amount of DNA, otherwise too much of the internal space is devoted to DNA storing and the remaining volume would not be sufficient for other cellular components (Trevors and Masson, 2011). Cell volume has a wide range of ∼0.013 to 400 μm^3^, however, a cell is considered small if its volume is less than 0.6 μm^3^ (Koch, 1996; Ghai et al., 2013). And third, the streamlining hypothesis suggests that the effects of economization are observable as an increase in fitness.

Therefore, genome reduction is believed to have had a favorable effect on fitness in *Prochlorococcus* species. In the oceanic ecosystem, the diversity of photosynthetic prokaryotes is mostly represented by two genera: *Prochlorococcus* and *Synechococcus.* While *Synechococcus* are ubiquitous owning to their flexibility and adaptability to various marine environments, the *Prochlorococcus* have had an apparently better ecological success in oligotrophic areas where the conditions are more stable (Partensky et al., 1999). The success of *Prochlorococcus* is believed to be due to the differential distribution across the vertical axis of the water column of specialized ecotypes which are genetically and physiologically distinct populations distributed in accordance with the quality of light (Scanlan et al., 2009).

Genome streamlining was suggested also to explain reductive genome evolution in the case of the α-proteobacterium “*Ca.* Pelagibacter ubique” HTCC1062 and other members from the SAR11 clade (Giovannoni et al., 2005; Grote et al., 2012). As described above, this proposal is also based on several features of its streamlined genome. For instance, “*Ca.* Pelagibacter ubique” was reported to have a median space size between coding genes of only three nucleotides, the smallest among then analyzed genomes. In addition, no pseudogenes, phage genes or recent gene duplications were found (Giovannoni et al., 2005). And similar to the case of *P. marinus* MED4 and SS120, it was suggested that the small cell size of “*Ca.* Pelagibacter ubique” (0.019–0.039 μm^3^) evolved by natural selection. In this case based on a theory proposed by Button (1991). Accordingly to this theory, selection optimized surface-to-volume ratio so that the capacity of the cytoplasm to process substrates matches transport rates (Giovannoni et al., 2005; Steindler et al., 2011).

The β-proteobacterias from Clade OM43 are another lineage where genome reduction by streamlining was suggested. As in the two cases described above, the small proportion of non-coding DNA in the reduced genome of strain HTCC2181 was interpreted as evidence of streamlining selection (Giovannoni et al., 2008). And as in the case of the strains MED4 and SS120 from *P. marinus* and in “*Ca.* Pelagibacter ubique,” the β-proteobacteria HIMB624 also has small cell size of about 0.1–0.3 μm wide and 0.6–1.8 μm long (Huggett et al., 2012).

Finally, streamlining could be suggested also for “*Ca*. Actinomarina minuta.” Its small genome is contained in an incredible small cell (0.013 μm^3^). The median length of its intergenic sequences is of only three bases, the same as of “*Ca.* Pelagibacter ubique.” “*Ca*. Actinomarina minuta” lives in aquatic environments where nutrients are scarce (Ghai et al., 2013).

Supporting the streamlining hypothesis, Koskiniemi et al. (2012) found that under laboratory conditions, selection can drive genome reduction. In order to do this, they devised a method that would report large deletions in the genome of *Salmonella enterica*. When they measured fitness against the wild type, they observed that several mutant strains showed an increase in fitness and concluded that fitness increases were common following deletions on specific genomic loci. Additionally, they performed a serial passage experiment and observed that selection could be a significant driver of gene loss. They suggested that in naturally occurring populations, fixation of deletion could occur very fast.

### ACCELERATED RATES OF PROTEIN EVOLUTION

Returning to *P. marinus* strains MED4 and SS120, accelerated rates of protein evolution has been observed in these cyanobacteria (Dufresne et al., 2005). This is similar to what is observed in symbiotic bacteria with reduced genomes (McCutcheon and Moran, 2012). However, the cause of this acceleration in free-living bacteria seems to be different. According to the streamlining hypothesis, this is a consequence of an increase in the mutation rate due to the loss of repair genes and not a direct consequence of selection. Supporting this hypothesis is the fact that both strains lack the *ada* gene, which encodes 6-*O*-methylguanine-DNA methyltransferase among other repair genes (Dufresne et al., 2005). In addition, the lack of this gene can lead to G:C to A:T transversions which, as discussed above, can be adaptive in a low phosphorus environments (Mackay et al., 1994; Dufresne et al., 2005). Nevertheless, differing from the marine picocyanobacteria described above, “*Ca.* Pelagibacter ubique” codes for the DNA repair enzyme 6-*O*-methylguanine-DNA methyltransferase while showing a G+C content as low as 29% (Giovannoni et al., 2005). This suggests that the loss of this enzyme is not a necessary prerequisite to evolve high levels of A+T and that the direct action of selection favoring high levels of A+T could be the cause.

### INCREASED RATE OF MUTATION HYPOTHESIS

An alternative explanation for genome reduction has been proposed which includes the previously mentioned accelerated rates of protein evolution for *P. marinus* strains (Marais et al., 2008). This explanation suggests that genome reduction occurs as a byproduct when an increased mutation rate becomes advantageous, like in the cases of novel niche colonization. And indeed, *P. marinus* MED4 and SS120 colonized high-light and low-light niches of the water column respectively between 150 and 80 million years ago (Dufresne et al., 2005).

**FIGURE 2:**
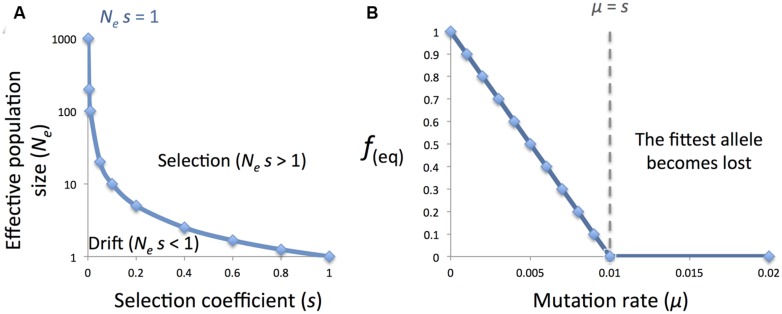
**Population genetic models. (A)** The fate of an allele is determined by natural selection if the product of the effective population size (*N_e_*) and the coefficient of selection (*s*) is larger than one, and by genetic drift otherwise; **(B)** however, when mutation is taken into account, the equilibrium frequency (*f_eq_*) of the fittest allele is 0 when the mutation rate (μ) is larger than the selection coefficient (*s*).

The argument is as follow. According to classical population genetic models, the fate of an allele is determined by selection if the product of the effective population size (*N_e_*) by the coefficient of selection (*s*) is larger than one (i.e., *N_e_* *s* > 1); and is determined by genetic drift if it is smaller than one: *N_e_* *s* < 1 (**Figure 2**; Gillespie, 1998). However, this model applies only when the mutation rate (μ) is negligible (Marais et al., 2008).

When mutation rate is not negligible and taken into account, the equilibrium frequency of the fittest allele becomes (1 μ/s) if μ < *s*, and 0 if μ > *s*. This is according to a simple model developed by Eigen (1971). Therefore, when the mutation rate is larger than the selection coefficient, the fittest allele will have an equilibrium frequency of 0 and therefore, will be lost in the population (Marais et al., 2008).

As mention above, high mutation rates can be advantageous when bacteria colonize new habitats. In natural populations some strains often develop increased mutation rates compared to the wild type due to the loss of repair genes. These strains are called mutator strains. Accordingly, mutator strains were selected during novel niche colonization by *P. marinus* MED4 and SS120. These increased μ over *s*, and favored the loss of genes that have only modest contribution to fitness thus reducing the genome (Marais et al., 2008).

In agreement with this hypothesis, the proteins from MED4 and SS120 *Prochlorococcus* have similar sizes to their homologs from *Prochlorococcus* with larger genomes. This is contrary to what would be expected if natural selection directly favored genome minimization as predicted by the streamlined theory (Marais et al., 2008). However, the same pattern can be accounted by the streamlining hypothesis if natural selection has not been strong enough to select for smaller proteins.

### THE BLACK QUEEN HYPOTHESIS

**FIGURE 3:**
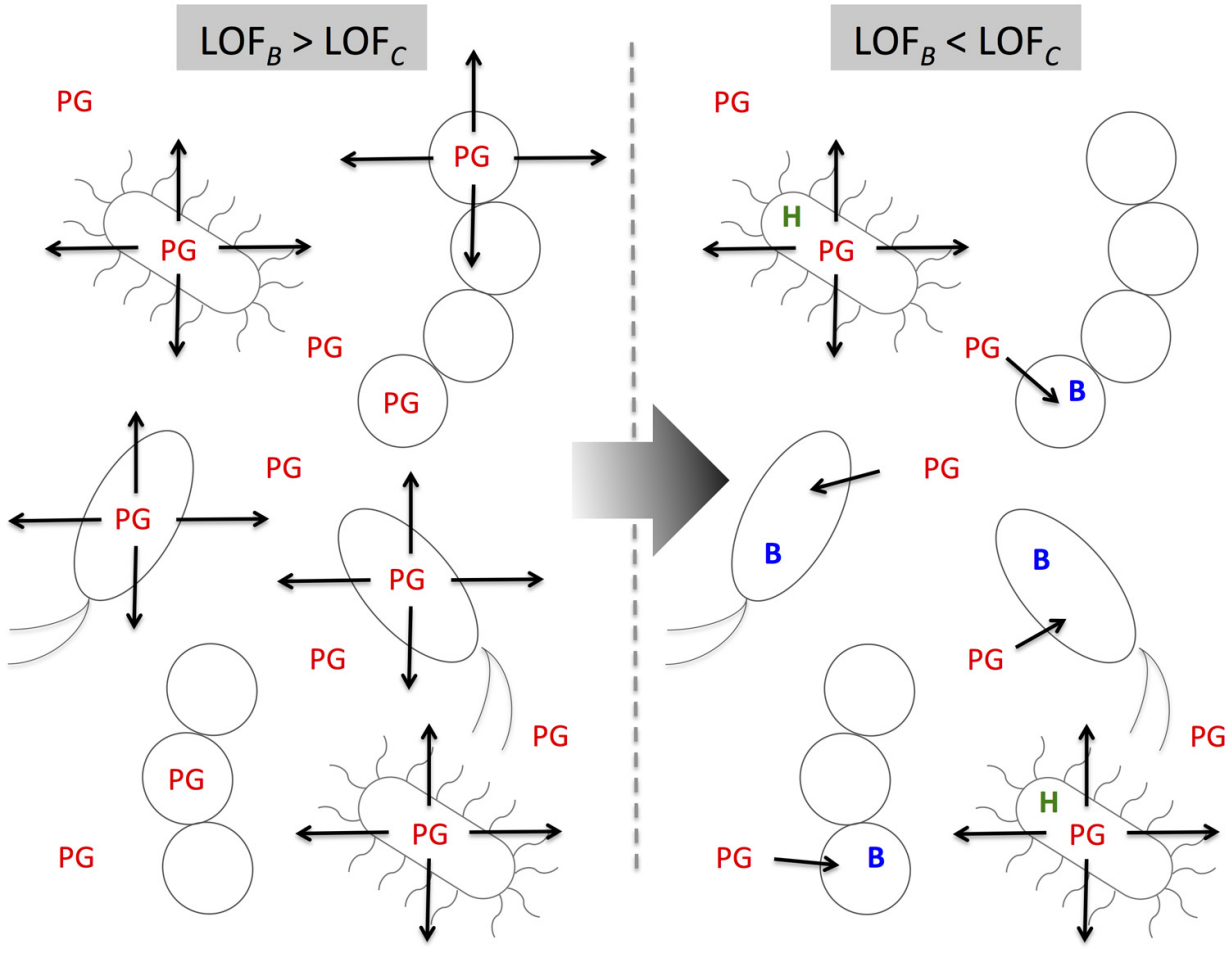
**The Black Queen Hypothesis predicts that if there is a community where different species produce an expensive and diffusible public good (PG), the system will evolve toward a scenario where only a few of their members will continue with the production of the PG, only if the benefit of losing the production of the function outweighs the cost of losing it.** In such community producers of the PG become helpers (H) and the rest become beneficiaries (B). LOF_B_, benefit of losing the function producing the PG; LOF_C_, cost of losing the function producing the PG.

Our discussion of the mechanisms of genome reduction would not be complete if we did not include a recent illuminating proposal known as the Black Queen Hypothesis (BQH; Morris et al., 2012). The BQH introduces gene loss and thus genomic reduction as a community-dependent adaptive event. In order for genomic reduction to occur according to the BQH there are three main components required in a community: a public good (PG), a helper and beneficiary organisms. A PG is a function or product that is energetically or nutritionally expensive to do or make, and that it is required and accessible by the whole community and not only by the producing organism. A helper organism is an organism capable of producing the PG and whether actively or passively is capable of leaking it to all the community. A beneficiary is an organism which utilizes the PG but becomes incapable of producing it itself. Genome reduction occurs in the beneficiary, and accordingly, there must be a selective advantage to lose the function and thus, the genes that code for it. Importantly, the benefit of losing the function is frequency-dependent, thus once the function becomes too scarce it is no longer advantageous to lose it, such that the presence of helpers is guaranteed in the population and permits the function to remain active for all the community (**Figure 3**; Morris et al., 2012).

As pointed out by Morris et al. (2012) and Sachs and Hollowell (2012), *Prochlorococcus* strains cannot grow axenically in its own habitat. This is because these bacteria lack the gene for catalase-peroxidase to eliminate hydrogen peroxide that is produced by photooxidation up to toxic levels by sunlight. For *Prochlorococcus* (a beneficiary) to grow under these conditions, it is necessary the presence of other bacteria (a helper) that code for catalase-peroxidase (a PG) to detoxify the environment. This is possible because hydrogen peroxide is permeable, thus the helper organisms catalyze the removal of hydrogen peroxide and reduces natural concentrations below the toxic level in marine environments (Morris et al., 2012).

Another example is that of highly reduced bacteria “*Ca*. Pelagibacter ubique.” Similarly as the above, this bacterium could not grow on artificial media until recently, when Carini et al. (2013) were capable of uncovering the mystery behind this organism amazing nutritional strategy. They found that this bacterium uses a balanced supply of organic matter, which included methionine, glycine, and pyruvate. These could be replaced by other metabolites, which included some common osmolytes. As such, they suggested that “*Ca*. Pelagibacter ubique” had evolved to efficiently utilize various low molecular weight metabolites of phytoplankton origin produced in low but continuous concentrations (Carini et al., 2013). Other suggested examples of genetic loss by BQH are related to nitrogen fixation, inorganic nutrient acquisition and biofilm matrix deposition but is likely that the list is much longer (Morris et al., 2012). Therefore, reductive genome evolution has to be re-analyzed at the light of the BQH. The community-dependent nature of the BQH would also suggest an important role in understanding the evolution of host-associated prokaryotes.

## HOST-ASSOCIATED PROKARYOTES WITH REDUCED GENOMES

Symbiosis can be defined as “*an intimate, close association between species in which the large majority or entire life cycle of one species occurs within or in very close association with another*” (Holland and Bronstein, 2008). As we mentioned earlier, symbiotic organisms tend to evolve smaller genomes amongst which host-associated intracellular mutualistic prokaryotes from different phyla have evolved the smallest cellular genomes known (**Table 1**). Although the term symbiosis is sometimes confounded with mutualism, this intimate and close association can be mutualistic or not. For intracellular prokaryotes these relationships are of parasitic, commensal as well as mutualistic nature. In particular, the biology of mutualistic prokaryotes with highly reduced genomes is strikingly diverse. Moreover, recent advances in genome sequencing have revealed novel and unexpected evolutionary trends, as briefly reviewed below.

**Table 1:** Mutualistic prokaryotes with reduced genomes.

Symbiotic prokaryotes	Taxonomy	Protein-coding genes	RNA-coding genes	Genome size (Kbp)		Host
*Nasuia deltocephalinicola* str. NAS-ALF *Sulcia muelleri* str. Sulcia-ALF	β-Proteobacteria Flavobacteriia	137 188	32 35	112 191	*Macrosteles quadrilineatus* (aster leafhopper)	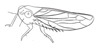

*“Ca.* Tremblaya princeps” PCVAL *“Ca.* Moranella endobia” PCVAL	β-Proteobacteria -Proteobacteria	116 411	20 47	139 538	*Planococcus citri* (citrus mealybug)	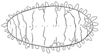

*“Ca.* Hodgkinia cicadicola” Dsem *“Ca.* Sulcia muelleri” SMDSEM	γ-Proteobacteria Flavobacteriia	169 242	19 33	144 277	*Diceroprocta semicincta* (cicada)	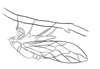

*“Ca.* Carsonella ruddii” PV	γ-Proteobacteria	182	31	160	*Pachypsylla venusta* (psyllid)	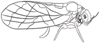

*“Ca.* Sulcia muelleri” GWSS *Baumannia cicadellinicola* Hc	Flavobacteriia γ-Proteobacteria	227 595	36 46	246 686	*Homalodisca coagulata* (sharpshooter)	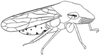

*“Ca.* Zinderia insecticola” CARI *“Ca.* Sulcia muelleri” CARI	β-Proteobacteria Flavobacteriia	202 246	29 34	209 277	*Clastoptera arizonana* (spittlebug)	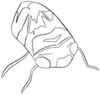

*“Ca.* Walczuchella monophlebidarum” Enterobacterial endosymbiont	Flavobacteriia γ-Proteobacteria	271¶ ns	36¶ ns	309 ns	*Llaveia axin axin* (scale insect “Niij”)	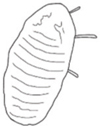

*“Ca.* Carsonella ruddii” CE Secondary endosymbiont of *Ctenarytaina eucalypti*	γ-Proteobacteria γ-Proteobacteria	190 918	31 45	163 1441	*Ctenarytaina eucalypti* (psyllid)	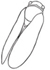

*“Ca.* Carsonella ruddii” HC Secondary endosymbiont of *Heteropsylla cubana*	γ-Proteobacteria y-Proteobacteria	192 576	31 44	166 1122	*Heteropsylla cubana* (psyllid)	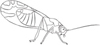

*“Ca.* Uzinura diaspidicola” ASNER	Flavobacteriia	227	35	263	*Aspidiotus nerii* Bouché (oleander scale)	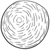

*“Ca.* Portiera aleyrodidarum” BT-QVLC *“Ca.* Hamiltonella defensa” MED	γ-Proteobacteria γ-Proteobacteria	246 1474	38 42	357 1843	*Bemisia tabaci* (Mediterranean whiteflies)	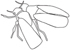

*Buchnera aphidicola* BCc *Serratia symbiotica* str. ‘Cinara cedri’	γ-Proteobacteria γ-Proteobacteria	362* 672	37 42	422* 1763	*‘Cinara cedri’* (cedar aphid)	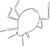

*Blattabacterium cuenoti* Cpu	Flavobacteriia	548*	38	610*	*Cryptocercus punctulatus* (wood roaches)	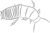

*“Ca.* Riesia pediculicola” USDA	γ-Proteobacteria	555*	40	582*	*Pediculus humanus corporis* (body louse)	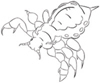

*Profftella armatura* *“Ca.* Carsonella ruddii” DC	β-Proteobacteria γ-Proteobacteria	372* 207	37 31	465* 174	*Diaphorina citri* (Asian citrus psyllid)	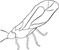

*Nanoarchaeum equitans* Kin4-M *Ignicoccus hospitalis* KIN4/I	Phylum: Nanoarchaeota Thermoprotei	540 1434	45 53	491 1298	*Ignicoccus hospitalis* KIN4/I	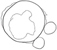

*Ishikawaella capsulata* Mpkobe	γ-Proteobacteria	623*	48	755*	*Megacopta punctatissima* (stinkbug)	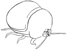

*Blochmannia vafer* BVAF	γ-Proteobacteria	587	42	723	*Camponotus vafer* (ant)	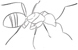

*Wigglesworthia glossinidia* endosymbiont of *Glossina morsitans* *Sodalis glossinidius* str. morsitans	γ-Proteobacteria γ-Proteobacteria	618 2516*	44 91	720 4293*	*Glossina morsitans* (tsetse fly)	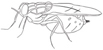

*“Ca.* Kinetoplastibacterium oncopeltii” TCC290E	β-Proteobacteria	694	52	810	*Strigomonas oncopelti* (tripanosome)	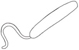

*“Ca.* Endolissoclinum faulkneri” L2	α-Proteobacteria	1498	48	1481	*Lissoclinum patella* (tunicate)	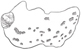

*“Ca.* Endomicrobium sp. Rs-D17”	Phylum: Elusimicrobia	776*	48	1149*	*Trichonympha agilis* (termite protist)	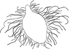

*“Ca.* Azobacteroides pseudotrichonymphae genomovar CFP2”	Bacteroidia	852*	42	1225*	*Pseudotrichonympha grassii* (termite protist)	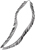

*Wolbachia* endosymbiont TRS of *Brugia malayi*	α-Proteobacteria	805	37	1080	*Brugia malayi* (filarial nematode)	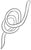

*“Ca.* Vesicomyosocius okutanii” Ha	γ -Proteobacteria	937	38	1022	*Calyptogena okutanii* (deep-sea clam)	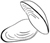

*“Ca.* Ruthia magnifica” str. Cm	γ-Proteobacteria	976	41	1161	*Calyptogena magnifica* (giant clam)	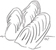

*Cyanobacterium* sp. UCYN-A	Subclass: Oscillatoriophycideae	1199	42	1440	Prymnesiophyte (unicellular eukaryote)	–

*“Ca.* Midichloria mitochondrii” IricVA	α-Proteobacteria	1211	38	1184	*Ixodes ricinus* (tick)	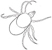

*Polynucleobacter necessarius* subsp. necessarius STIR1	β-Proteobacteria	1279^†^	44	1560	*Euplotes aediculatus* (ciliated protist)	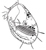

The information of genes and gene size was obtained from Genbank. The class is shown in taxonomy unless specified. “ns”: values unknown from not-sequenced organism. “*”: Values include the gene content and genomic size of strain specific plasmids. “¶”: Values as reported in the original article (Rosas-Pérez et al., 2014). “: Value as reported in the original article (Boscaro et al., 2013). Host drawings are kindly provided by Sofia Delaye.

### EARLY OBLIGATED INTRACELLULAR SYMBIOSIS

The transition from free-living to endosymbiotic mutualistic lifestyle was recently studied by comparative genomics of free-living bacteria and their counterparts, living either in a protist or in insects (weevils or aphids; Clayton et al., 2012; Boscaro et al., 2013; Manzano-Marín and Latorre, 2014; Oakeson et al., 2014). These studies showed that in both cases, gene inactivation, genome rearrangement and loss of some of the repair mechanisms played important roles. However, there were also important differences. In particular, there is an extreme proliferation of MGEs in symbiotic bacteria associated to the early stages of the integration process in insects, but not in the bacteria associated to the protist. This difference was attributed to the fact that in the case of the insect, the population of the bacteria undergoes recursive bottlenecks that lower the efficacy of selection allowing the proliferation of MGEs (Mira et al., 2001). Furthermore, the importance of mobile elements in the transition from free-living to an obligate endosymbiotic state involves their participation in gene inactivation and genome size reduction in recent endosymbiont genomes, as observed from comparative studies between ancient endosymbionts having lost all mobile elements, and related free-living bacteria, with a controlled number of these, presumably by natural selection (Manzano-Marín and Latorre, 2014; Oakeson et al., 2014).

Observational as well as experimental data indicate that genome reduction in host-associated bacteria occurs at a fast rate once the symbiosis is obligate. For instance, it is estimated that the obligate endosymbiont of the rice weevil *Sitophilus oryzae*, “*Candidatus* Sodalis pierantonius” str. SOPE, lost 55% of their genes in just 28,000 years (Oakeson et al., 2014). In agreement with previous observation, experimental evidence shows that under laboratory conditions, similar to those of intracellular bacteria (strong bottlenecks and absence of HGT), genome reduction can occur very rapidly on an evolutionary time scale (Nilsson et al., 2005).

“*Candidatus* Sodalis pierantonius” str. SOPE lives inside the rice weevil’s bacteriocytes and has a relatively large genome coding for about 2,309 protein-coding genes and 1,771 pseudogenes. Perhaps the most striking characteristic of the genome of this bacterium is that it is plagued with MGEs, about 18% of the genome consists of ISs. These MGEs have contributed in this organism to genome rearrangement, partial genome duplications, deletogenic rearrangements and to ∼10% of gene inactivation. This is dramatically exemplified by the constant perturbations of the G+C skew in its chromosome that reveals multiple changes in leading versus lagging strand orientation (Oakeson et al., 2014).

Early evolution of intracellular mutualistic symbiosis was also studied in aphids. Manzano-Marín and Latorre (2014) compared the genome of bacteria in different stages of the process of adaptation to intracellular life-style. This comparison included three strains of “*Candidatus* Serratia symbiotica” which represented an early facultative stage (“*Ca.* Serratia symbiotica” from *Acyrthosiphon pisum,* SAp) a later facultative bordering on early obligate stage (“*Ca.* Serratia symbiotica” from *Cinara tujafilina,* SCt), and an co-obligate stage (“*Ca.* Serratia symbiotica” from *Cinara cedri,* SCc). Strain SCc has obligate endosymbiotic characteristics, such as the lack of MGEs, high A+T content and no genetic redundancy. However, it possesses large intergenic regions, remnants of ancient pseudogenes still not degraded (Lamelas et al., 2011a). On the other hand strain SCt, while being phylogenetically and genomically very closely related to the facultative *SA*p strain (Burke and Moran, 2011), it shows a variety of metabolic, genetic and architectural features which point toward this endosymbiont being one step closer to an obligate intracellular life-style. By studying the genome rearrangements and the impact that MGEs have had on the genome architecture of these two *Serratia* endosymbionts (SCt and SAp), it was determined that those genes belonging to IS families have been the key factor promoting massive rearrangements. These MGEs have also mediated inactivation in various genes, sometimes creating long stretches of inactivated proteins in tandem (Manzano-Marín and Latorre, 2014).

In the case of bacteria associated to the protist, the genome sequences of free-living and symbiotic strains of the β-proteobacteria *Polynucleobacter necessarius* were compared. The free-living strain is a common inhabitant of lentic freshwater ecosystems, while the symbiotic strain lives as an intracellular symbiont of the ciliated protist *Euplotes aediculatus*. These strains diverged very recently, as shown by their similarity at the level of the 16S rRNA which is >99% (Boscaro et al., 2013). The genome of the free-living strain contains 2,088 protein-coding genes (Meincke et al., 2012) and is itself a reduced genome (Boscaro et al., 2013). The genome of the symbiotic strain contains 1,279 protein-coding genes and is mostly a subset of the free-living strain (i.e., only 105 genes are not shared with its free-living relative). The symbiotic strain also contains between 231 and 460 pseudogenes. Of course, the ultimate cause of this genome reduction is the endosymbiotic lifestyle of *P. necessarius*. However, the proximal cause of the genome reduction is less well understood. Furthermore, the metabolic bases of the obligate symbiosis between the bacteria and the ciliate are not known. However, it was suggested that *P. necessarius* complements some metabolic deficiency in *E. aediculatus* (Boscaro et al., 2013). Nevertheless, it was suggested that genome reduction in the symbiotic strain was caused by illegitimate recombination and loss of mismatch repair genes. In addition it was suggested that the early loss of the gene coding for the translesion DNA polymerase exerted further evolutionary pressure for a smaller genome and favored polyploidy, which is one of the main differences between both strains such that the symbiotic strain contains several nucleoids, each one containing one copy of the genome (Boscaro et al., 2013).

As exemplified by *P. necessarius,* rapid gene loss can occur in the absence of proliferation of MGEs. The endosymbiotic strain of *P. necessarius* has already lost over 40% of its coding capacity with 13–18% still observable as pseudogenes. This loss occurred in the absence of MGEs, and as mentioned above, the lack of MGEs in *P. necessarius* has been attributed to a larger population size relative to that of “*Ca*. Sodalis pierantonius” str. SOPE (Boscaro et al., 2013).

In addition to the previously mentioned examples, the lack of proliferation of MGEs in the case of *P. necessarius* contrast with other obligate symbiosis. These include *Burkholderia rhizoxinica* the endosymbiont of the fungus *Rhizopus microsporus* with 6% of their encoded proteins similar to transposases (Lackner et al., 2011a); the γ1 symbiont of the marine oligochaeta *Olavius algarvensis* that has a genome coding for 20% of transposases (Woyke et al., 2006); NoAz, the extracellular mutualistic endosymbiotic cyanobacteria of the water-fern *Azolla filiculoides* with ∼600 IS elements (Ran et al., 2010); and the facultative symbiont of the whitefly “*Candidatus* Cardinium hertigii” possessing ∼200 MGEs (Santos-Garcia et al., 2014).

Proliferation of MGEs can occur also in the absence of massive genome rearrangement, as shown in the case of the intracellular bacteria “*Candidatus* Amoebophilus asiaticus” that maintains a regular G+C skew along its genome despite that 24% of its genes code for MGEs (Schmitz-Esser et al., 2010, 2011). Nevertheless, this last comparison has to be taken with caution since “*Ca.* Amoebophilus asiaticus” has not adapted recently to the intracellular lifestyle, has received several genes by HGT and as a parasite is under different evolutionary pressures than mutualistic endosymbionts (Schmitz-Esser et al., 2010).

### PARADOXICALLY LARGE G+C CONTENT IN TWO HIGHLY REDUCED GENOMES

As in the case of free-living bacteria with reduced genomes, host obligated bacteria with reduced genomes also show large levels of A+T content (Moran, 2003; Moya et al., 2008; Delaye et al., 2010). It is hypothesized that the loss of repair enzymes due to genome reduction, in combination with a reduced efficacy of natural selection, and a universal G:C to A:T mutational bias in bacteria, is responsible of the observed trend (Hershberg and Petrov, 2010; Hildebrand et al., 2010; Van Leuven and McCutcheon, 2012).

However, there are notable exceptions to the above rule. The cicada *Diceroprocta semicincta* contains in its bacteriome two symbiotic bacteria, the α-Proteobacteria “*Candidatus* Hodgkinia cicadicola” and the Flavobacteria “*Candidatus* Sulcia muelleri” (McCutcheon et al., 2009a,b). The genome of “*Ca.* Hodgkinia cicadicola” is in itself a paradox for molecular evolutionary theory because it has relatively large G+C content (∼58%) despite having an extremely small genome (∼144 kbp). This high G+C content is not due to biased gene conversion, since this bacterium lacks repair enzymes. Also, the high G+C content is not due to an A:T to G:C mutational bias since it is known that “*Ca.* Hodgkinia cicadicola” suffers from the same G:C to A:T mutational pressure universally present in bacteria (Van Leuven and McCutcheon, 2012).

One possibility that was suggested to explain this unexpectedly large G+C content is that the demography of its host *D. semicincta* inflates the population size of “*Ca.* Hodgkinia cicadicola” making natural selection more efficient to counter balance the G:C to A:T mutational bias (Van Leuven and McCutcheon, 2012). However, it is not clear what benefit could confer single G:C over A:T polymorphisms in these bacteria. Other possibilities are selection for better DNA replication and/or DNA packing on C+G rich genomes (Hershberg and Petrov, 2010). A similar situation is found in “*Candidatus* Tremblaya princeps,” symbiont of the citrus mealybug *Planococcus citri*, which also shows an extremely reduced genome of ∼139 kbp and a relatively high G+C content (McCutcheon and von Dohlen, 2011; López-Madrigal et al., 2013b).

### UNEXPECTED LOSS OF GENOMIC STABILITY

During the early stages of reduction, genome architecture is quite unstable as discussed above. Many rearrangements occur over relatively short periods of time. As an example of this, the comparison of the genome architectures of six free-living *Serratia* and three “*Ca.* Serratia symbiotica” endosymbionts, showed surprising amounts of genomic rearrangements suffered between the three *S. symbiotica* lineages (Manzano-Marín and Latorre, 2014).

However, as the endosymbiosis evolves toward the last stages of reduction, genomic stability increases to a stalemate such that gene order conservation was considered one of the hallmarks of the genomes of obligate mutualistic bacteria. This was first noticed among genomes of *Buchnera aphidicola* from different strains with more than 50 million years of divergence. Initially, this high degree of conservation in gene order was attributed to absence of the *recA* gene in these bacteria. Its product, RecA, is the key enzyme in homologous recombination repair (Shigenobu et al., 2000; Tamas et al., 2002; van Ham et al., 2003).

Homologous recombination is a high-fidelity DNA repair mechanism for double strand break. The broken DNA is processed at the ends by several possible pathways producing a 3′-tailed duplex onto which RecA is loaded. RecA is an ATP-dependent multifunctional enzyme, which has recombinase activity. RecA assembles into a filament and then sequesters the template double stranded DNA, where it looks for the homologous loci, exchanges DNA strands and forms joints between recombining molecules which allows the recombination and repair of the broken chromosome (Spies, 2013; Wigley, 2013).

However, the hypothesis that genome stasis is the result of loss of *recA* lost support when it was found that genome sequences from other endosymbionts like *Blattabacterium*, *Carsonella,* and *Wigglesworthia* showed similar levels of synteny conservation despite coding for *recA* (reviewed in Sloan and Moran, 2013). Additionally, recent genome sequencing projects showed that lack of repair and recombination genes may not be the cause of genome stability. “*Candidatus* Portiera aleyrodidarum” the primary endosymbiont of whiteflies (*Bemisia tabaci*) shows genome structural polymorphisms (i.e., lack of synteny) despite lacking *recA* and having one of the most reduced repair and recombination gene sets. These polymorphisms are demonstrated to be present even within bacteria inhabiting individual hosts and likely within individual bacterial cells. The presence of such structural polymorphisms was attributed to recombination events between large intergenic regions and repetitive elements that in turn are maintained by gene conversion (Sloan and Moran, 2012b, 2013).

A similar case of loss of genome stability was found in “*Ca.* Tremblaya princeps.” This bacterium shows a 7,032 bp region flanked by inverted repeats that is found in both orientations in the population. And similar to “*Ca.* Portiera aleyrodidarum,” this bacterium also codes for a highly reduced set of DNA replication, recombination and repair enzymes (McCutcheon and von Dohlen, 2011; López-Madrigal et al., 2013b).

### NOVEL HYPOTHESIS TO EXPLAIN THE REASSIGNMENT OF STOP TO Trp CODON

The obligate endosymbionts “*Candidatus* Nasuia deltocephalinicola,” “*Candidatus* Zinderia insecticola,” and “*Ca.* Hodgkinia cicadicola” evolved an alternative genetic code in which the codon UGA is reassigned from coding to stop (UGA_stop_) to code for tryptophan (UGA_Trp_). This codon reassignment has been observed also in mycoplasmas and some mitochondrial genomes (McCutcheon et al., 2009a; Bennett and Moran, 2013).

**FIGURE 4:**
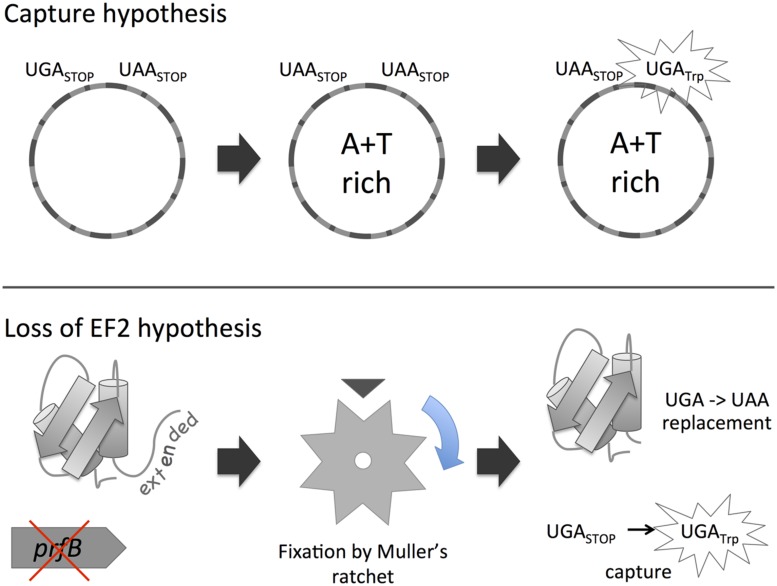
**The reassignment of stop codon (UGA_**stop**_) to code for tryptophan (UGA_**Trp**_) is explained using the “capture hypothesis.”** However, the large G+C content in “*Ca*. Hodgkinia cicadicola” makes the capture hypothesis unlikely in this organism. Instead, it is hypothesized that the lose of translational release factor RF2 triggered the evolution of this rearrangement.

The evolution from UGA_stop_ to UGA_Trp_ has been explained with the “capture” hypothesis (**Figure 4**). According to this model, all UGA codons mutate first to its synonymous codon UAA in A+T rich genomes. This change does not affect protein length or fitness. Then, when UGA re-appears through mutation, it is free to be “captured” by an amino acid, in this case Trp. The fact that almost all reassignments of the UGA_stop_ to UGA_Trp_ evolved in A+T rich genomes supports this hypothesis (Osawa and Jukes, 1989).

Nonetheless, “*Ca*. Hodgkinia cicadicola” has a relatively high G+C content of about 58.4% making the “capture” hypothesis an unlikely explanation for this phenomenon. Therefore, a new hypothesis was proposed to explain the reassignment of UGA_stop_ to UGA_Trp_ in this bacterium. The new hypothesis proposes that the loss of translational release factor RF2 (encoded by *prfB*) that recognizes UGA as a stop codon triggered the evolution of this rearrangement (McCutcheon et al., 2009a). According to this scenario, the loss of *prfB* caused that some proteins were translated with an extended sequence. These “extended” proteins were not necessarily lethal, perhaps just slightly deleterious. This is supported by experiments showing that extended proteins can increase fitness under stress conditions in yeast (Halfmann et al., 2012). Since symbiotic bacteria from insects are subject to recurrent bottlenecks, these slightly deleterious mutations become fixed by genetic drift under a Mullerian ratchet process. Then, once they are fixed, natural selection favored replacement of UGA_Stop_ codons with functional UAA or UAG, thus restoring the original size of proteins. This in turn allows UGA to be captured by tRNA-Trp (McCutcheon et al., 2009a).

### CLUES TO EARLY LIFE?

The genome of “*Candidatus* Riesia pediculicola” the symbiont of the body louse *Pediculus humanus corporis*, codes for what seems to be a minimal tRNA decodification set (Kirkness et al., 2010). This endosymbiont lost all the enzymes that modify the tRNA body, and kept only those genes that make modifications of the anticodon-stem-loop which are essential for mRNA decoding. Therefore, it was suggested that the minimal tRNA decodification set of this bacterium could resemble that of very ancient cells that existed during the early evolution of life on Earth (Kirkness et al., 2010). In support of this hypothesis, pseudouridine synthase A (encoded by *truA*), which is the enzyme responsible of the formation of pseudouridine at positions 38, 39, and 40 in the anticodon stem loop of tRNAs was suggested to be present in the last common ancestor of all extant life, or *cenancestor* (Ouzounis et al., 2006). However, at this moment, there is no evidence that the rest of the enzymes of the tRNA codification set of “*Ca.* Riesia pediculicola” are as ancient as *truA*. Nevertheless, this tRNA modification set exemplifies how simpler cellular systems can work. Which is highly relevant for synthetic biology approaches to the minimal cell. Whether other symbionts offer clues to early life on Earth still has to be carefully discussed.

### THE ROLE OF HORIZONTAL GENE TRANSFER IN THE EVOLUTION OF INTRACELLULAR SYMBIOSIS

One of the most striking peculiarities of host-associated bacteria with reduced genomes is how these organisms perform their symbiotic function and all the necessary process to maintain themselves with such a reduced gene set. There are at least three non-mutually exclusive possibilities (McCutcheon and von Dohlen, 2011). On the first place, modifications of some genes coded in the reduced genome could allow the endosymbiont to cope with the loss of otherwise essential genes; second, the presence of complementary genes in the genomes of co-symbionts (if any) may compensate for gene losses in the endosymbiont; and third, genes coded in the genome of the host compensate for gene losses in the genome of the endosymbiont. From an evolutionary point of view, this last group of genes could be of host origin, or originally from the endosymbiont and transferred to the host, or horizontally transferred from unrelated organisms not participating in the symbiosis to the host genome or its endosymbionts (McCutcheon, 2010; McCutcheon and von Dohlen, 2011).

Horizontal gene transfer is one of the main forces in prokaryotic evolution (Zhaxybayeva and Doolittle, 2011). Recent discoveries show that HGT has played a role in the evolution of some obligate mutualistic symbiosis. For instance, “*Ca.* Carsonella ruddii” the obligate symbiont from the psyllid *Pachypsylla venusta* has one of the smallest genomes with 213 genes and ∼160 kbp. This organism lives in the absence of other co-symbionts (Nakabachi et al., 2006). And as expected for such a small genome, a detailed analysis of its gene content indicated that several functions considered essential for a cell are missing (Tamames et al., 2007), raising the question of how these bacteria accomplish its symbiotic function. A recent transcriptomic analysis showed that the biosynthesis of essential and non-essential amino acids is performed collaboratively by the symbiont and by genes expressed in the bacteriocytes some of them of bacterial origin, and at least one of them directly acquired from “*Ca*. Carsonella ruddii” (Sloan et al., 2014).

Similarly, the genome of the pea aphid (*A. pisum*) does contain genes of bacterial origin that are highly expressed in the bacteriocytes and likely participate in the symbiosis with *B. aphidicola* Aps (Nikoh and Nakabachi, 2009; The International Aphid Genomics Consortium, 2010). These functional genes in *A. pisum* were acquired from bacteria other than its primary endosymbiont *B. aphidicola* Aps (Nikoh et al., 2010). Noteworthy, it was recently shown that the protein RplA4, coded by one of these genes, is targeted to the cytoplasm of the *B. aphidicola* Aps (Nakabachi et al., 2014). A finding that has been interpreted as blurring the distinction between endosymbionts and organelles (McCutcheon and Keeling, 2014). In addition, the only genes of *B. aphidicola* Aps origin in the genome of *A. pisum* are two highly truncated pseudogenes (Nikoh et al., 2010). Furthermore, experimental evidence has shown that the biosynthesis of some of the essential amino acids provided by *B. aphidicola* is performed partially by host enzymes (Russell et al., 2013).

Another case is found in the citrus mealybug *P. citri* where at least six distinct lineages of bacteria contributed with horizontally transferred genes to its nucleus. These genes code for protein products that complement the biosynthesis of essential amino acids, vitamins and peptidoglycan in their endosymbionts “*Ca*. Tremblaya princeps” and “*Candidatus* Moranella endobia” (Husnik et al., 2013).

Also, HGT contributed to the acquisition of toxicity in other symbiotic systems. The genome of “*Candidatus* Profftella armatura” the symbiont of the Asian citrus psyllid (*Diaphorina citri*) acquired by HGT genes for the synthesis of cytotoxic polyketides. In this tripartite symbiosis, “*Ca*. Profftella armatura” produces the polyketides, while another bacterium from the genus *Carsonella* provides the host with essential amino acids (Nakabachi et al., 2013).

The recent genome sequencing of the filarial nematode *Brugia malayi* showed that approximately 10.6% of the genome of its symbiont, *Wolbachia* wBM has been transferred to the eukaryotic genome. Interestingly, there is evidence that some of the genes coded in these regions are transcribed in particular stages of the life cycle of the nematode suggesting functionality. However, their role in symbiosis still has to be determined (Ioannidis et al., 2013).

And finally, the synthesis of essential amino acids in symbiont harboring trypanosomes is carried in part by genes of bacterial origin coded in the genome of the protist (Alves et al., 2013). These data clearly shows that HGT is an important force in the evolution of some intracellular symbiosis (Sloan et al., 2014).

However, not all the symbiosis shows evidences of HGT. The genome sequence of the body louse *P. h. corporis* does not appear to contain any genes of prokaryotic origin, indicating no transfer from its endosymbiont “*Ca.* Riesia pediculicola” strain USDA, nor other bacteria (Kirkness et al., 2010). Similarly, “*Candidatus* Endolissoclinum faulkneri” a defensive symbiont that also produces cytotoxic polyketes and that inhabits *Lissoclinum patella*, a colonial filter-feeding tunicate, does not seem to have acquired this capacity through HGT (Kwan et al., 2012).

### BIOCHEMICAL COMPLEMENTARITY AND CONVERGENT EVOLUTION OF CO-RESIDENT SYMBIONTS

In cases where there are more than one species of obligate mutualistic symbiont, the biosynthesis of relevant metabolites for the host often requires the participation of enzymes that are coded in both co-symbionts and in some cases, as reviewed above, in the insect host (McCutcheon and von Dohlen, 2011). Furthermore, there are some occasions where different symbiotic systems conformed by two co-symbionts have converged independently to the same division of labor regarding the biosynthesis of amino acids that are provided to their hosts.

**FIGURE 5:**
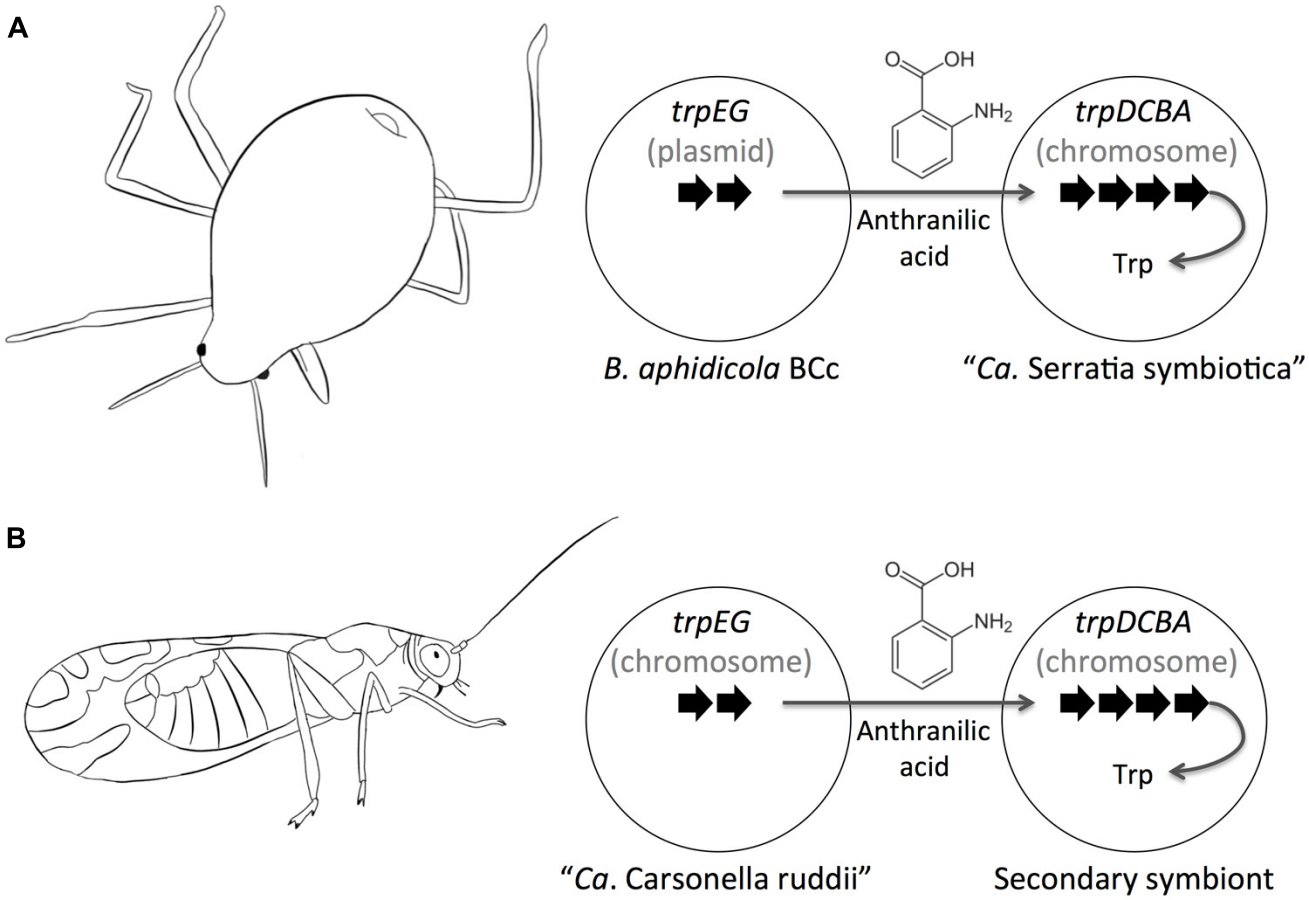
**Convergent evolution of Trp biosynthesis.** The biosynthesis of Trp is performed cooperatively by two different endosymbionts in the aphid *Cinara cedri* **(A)** and in the psyllid *Heteropsylla cubana* **(B)**. Strikingly, the same division of labor evolved in both systems.

In this sense *B. aphidicola* BCc, from the aphid *C. cedri*, requires the co-symbiont “*Ca.* Serratia symbiotica” to provide Trp to its host. In this symbiotic system, the first two genes of Trp biosynthesis (*trpEG*) are located in a plasmid in *B. aphidicola* BCc, while the rest of genes (*trpDCBA*) are located on the main chromosome of “*Ca.* Serratia symbiotica” (**Figure 5**; Pérez-Brocal et al., 2006; Gosalbes et al., 2008). The same phenomena occurs in the symbiotic system of the psyllid *Heteropsylla cubana*, where one of the symbionts (tentatively classified as a secondary symbiont) lost nearly all genes for the biosynthesis of essential amino acids except those of the (*trpDCBA*) operon, however, complementing the Trp biosynthetic capabilities of “*Ca*. Carsonella ruddii” HC (Sloan and Moran, 2012a). As exemplified above, the secondary symbiont of *H. cubana* and “*Ca.* Serratia symbiotica” have both evolve convergently to code for the same genes required for the biosynthesis of Trp (*trpDCBA*).

A similar biosynthesis of Trp is observed in the case of *B. aphidicola* BCt, the symbiont of the aphid *C. tujafilina*, where the first two genes of of Trp biosynthesis (*trpEG*) are also located in a plasmid together with the structural genes for leucine synthesis. However, differing from *B. aphidicola* BCc, the biosynthesis of Trp does not occur cooperatively between two symbionts since the rest of the genes of Trp biosynthesis are located in the main chromosome of *B. aphidicola* BCt (Gil et al., 2006; Lamelas et al., 2011b).

Another remarkable case of metabolic complementarity is provided by the co-symbionts *Baumannia cicadellinicola* and “*Ca.* Sulcia muelleri” Hc from the xylem-feeding glassy-winged sharpshooter *Homalodisca coagulata*. For instance, *B. cicadellinicola* is able to make cysteine from homoserine and coenzyme A from 2-ketovaline, but is unable to make homoserine and 2-ketovaline. And “*Ca.* Sulcia muelleri” Hc is able to make homoserine from aspartate and 2-ketovaline by the valine biosynthetic pathway. This kind of complementarity extends also to the biosynthesis of fatty acids, where *B. cicadellinicola* has all the genes necessary for the synthesis of fatty acids except for *fabF*, which in turn is encoded in “*Ca.* Sulcia muelleri” Hc. However, it is not clear how these molecules are transported across membranes since “*Ca.* Sulcia muelleri” Hc possesses few transporters (McCutcheon et al., 2009b).

Additionally, “*Ca.* Sulcia muelleri” Hc provides its host with eight of the ten essential amino acids (arginine, phenylalanine, tryptophan, lysine, threonine, isoleucine, leucine, and valine) required by the hosts, while *B. cicadellinicola* produces the remaining two which are methionine and histidine. At the same time, the same scheme of amino acid provision by co-symbionts is found in the cicada *D*. *semicincta*. Both, *D*. *semicincta* and *H. coagulata* share the endosymbiont “*Ca.* Sulcia muelleri” which provides the same eight essential amino acids. However, *H. coagulata* and *D. semicincta* differ in their co-symbiont accompanying “*Ca.* Sulcia muelleri.” While *H. coagulata* contains *B. cicadellinicola*, *D. semicincta* hosts “*Ca*. Hodgkinia cicadicola.” This suggest that *B. cicadellinicola* and “*Ca*. Hodgkinia cicadicola” have evolved convergently to provide methionine and histidine to their host (Wu et al., 2006; McCutcheon et al., 2009b).

It is important to note that, as expected from convergent evolution, there are metabolic differences between *B. cicadellinicola* and “*Ca.* Hodgkinia cicadicola” particularly in the biosynthesis of methionine. While *B. cicadellinicola* uses the cobalamin (vitamin B12)-independent version of methionine synthase, “*Ca.* Hodgkinia cicadicola” uses the cobalamin-dependent version of the enzyme. Both bacteria differ as well on the vitamin and cofactor biosynthetic capabilities (McCutcheon et al., 2009b).

In a similar fashion, the spittlebug *Clastoptera arizonana* contains in its bacteriome “*Ca.* Sulcia muelleri” CARI and “*Ca.* Zinderia insecticola” (McCutcheon and Moran, 2010). And as in the cases described above, both kinds of bacteria are needed to provide with the ten essential amino acids to their host. However, in this case “*Ca.* Sulcia muelleri” CARI cannot make Trp. Instead, this amino acid is synthetized by “*Ca.* Zinderia insecticola” in addition to methionine and histidine (McCutcheon and Moran, 2010).

Finally, the phloem-feeding aster leafhopper (ALF) *Macrosteles quadrilineatus* contains in separated cells within its bacteriome the symbionts “*Ca.* Sulcia muelleri*”* ALF and “*Ca.* Nasuia deltocephalinicola” ALF (Bennett and Moran, 2013). The genome of “*Ca.* Sulcia muelleri*”* ALF with 190,733 bp and 188 protein-coding genes is the smallest among sequenced genomes from this genus of bacterial symbionts. And the genome of “*Ca*. Nasuia deltocephalinicola*”* ALF with 112,091 bp and 137 protein-coding genes is the smallest bacterial genome sequenced so far. Similarly as above, “*Ca.* Sulcia muelleri*”* ALF codes for the genes necessary to synthetize the same eight essential amino acids and “*Ca*. Nasuia deltocephalinicola*”* ALF codes for the genes required to produce methionine and histidine. (Bennett and Moran, 2013).

It seems that “*Ca*. Sulcia muelleri” infected the ancestor of a large group of sap-feeding insects (Auchenorrhyncha) including the sharpshooter and cicada >260 million years ago (McCutcheon and Moran, 2007). This is clearly seen by the fact that the genomes of “*Ca*. Sulcia muelleri” from *D*. *semicincta* and *H*. *coagulata* are almost collinear despite having diverged several million years ago (McCutcheon et al., 2009b).

Interestingly, *Nasuia* and *Zinderia* appear to be sister clades, which suggested the existence of an ancient lineage of β-proteobacterial endosymbionts hosted at least since the divergence of Cicadomorpha from Fulgoroidea 200 million years ago which are the only two clades in the suborder Auchenorrhyncha. This also suggests the loss and latter independent acquisition of *B*. *cicadellinicola* and “*Ca*. Hodgkinia cicadicola” in the lineage leading to the sharpshooter and the cicada, respectively. The exchange in hosted symbionts was hypothesized to correlate with the new nutritional needs related to the diversification in the host diet (Bennett and Moran, 2013).

Similarly, metabolic complementation is suggested for the amino acid biosynthesis in the flavobacterium “*Ca*. Walczuchella monophlebidarum*”* where it was observed that many missing genes and pseudogenes in “*Ca*. Walczuchella monophlebidarum*”* were present in the γ-proteobacterial (Enterobacteriaceae) co-symbiont (Rosas-Pérez et al., 2014). Similarly as observed in the co-speciation between “*Ca*. Sulcia muelleri” and sap-feeding insects, co-speciation was observed between Flavobacteria and several scale insects but not so with the Enterobacteriaceae symbiont suggesting a similar trend in metabolic complementarity (Rosenblueth et al., 2012).

Metabolic complementarity is also observed in the obligate symbionts of the Asian citrus psyllid *D. citri* which are “*Ca.* Carsonella ruddii*”* DC and “*Ca*. Profftella armatura.” The metabolic capacities of both symbionts are largely non-redundant. For instance, the genome of “*Ca*. Profftella armatura” encodes 16 genes for coenzyme transport and metabolism, including those for the synthesis of riboflavin and biotin, and the genome of “*Ca.* Carsonella ruddii*”* DC completely lacks these genes (Nakabachi et al., 2013).

Another extraordinary case of metabolic convergence is provided by *Blattabacterium* strain BGe that is the primary endosymbiont of the German cockroach *Blattella germanica*, and the carpenter ant endosymbionts from the genus *Blochmannia* spp. (López-Sánchez et al., 2009). While *Blattabacterium* strain BGe is a member of the Bacteroidetes, *Blochmannia* spp. belongs to Proteobacteria. Despite the different phylogenetic origin of these bacteria, they resemble each other at the broad level of functional gene categories. A situation not found when *Blattabacterium* is compared with other insect endosymbionts like *Wolbachia* sp. and *Sulcia muelleri*. It seems that both bacteria converged due to the omnivorous diets of their hosts (Feldhaar et al., 2007; Patiño-Navarrete et al., 2014).

Finally, and perhaps one of the most striking examples of complementary is provided by one of the most extreme cases of symbiosis in nature. “*Ca*. Tremblaya princeps,” a prokaryote with one of the smallest genomes, that as described above, lives inside the bacteriocytes of the mealybug *P*. *citri* and contains itself the bacteria “*Ca*. Moranella endobia” (McCutcheon and von Dohlen, 2011; López-Madrigal et al., 2013b). In “*Ca.* Tremblaya princeps” most of its genes are devoted to RNA metabolism, the assembly of iron–sulfur [Fe–S] clusters and to the partial biosynthesis of some essential amino acids. However, its genome does not code for any complete pathway. Therefore, “*Ca.* Tremblaya princeps” seems to depend for almost all basic functions on the coding capacities of “*Ca*. Moranella endobia” and likely from host-encoded proteins. In fact, it was proposed that “*Ca.* Tremblaya princeps” acquires the necessary cell components to function by sporadic cell lysis of “*Ca*. Moranella endobia” (McCutcheon and von Dohlen, 2011). The distribution of coded tRNAs in the genomes of “*Ca.* Tremblaya princeps” and “*Ca*. Moranella endobia” supports this hypothesis (López-Madrigal et al., 2013b). However, immunohistochemistry assays with polyclonal antibodies to identify the location of the channel protein MscL coded only by “*Ca*. Moranella endobia” and GroEL coded by both bacteria, did not find evidence of massive and constitutive protein movement from the cytoplasm of “*Ca*. Moranella endobia” to the cytoplasm of “*Ca.* Tremblaya princeps” (López-Madrigal et al., 2013a).

### GENOME REDUCTION IN BACTERIAL SYMBIONTS OF FUNGI, A RELATIVELY UNEXPLORED WORLD

Bacteria and fungi are commonly co-inhabitants of a large variety of niches on Earth. Both groups of microorganisms are the major colonizers of terrestrial and aquatic environments, and both have been revealed as intracellular guests of eukaryotic hosts (Bonfante and Genre, 2008; Moran et al., 2008; Gibson and Hunter, 2010). Despite the fact that bacterial-fungal interactions are ubiquitous and relevant for the industry, agriculture and medicine (Frey-Klett et al., 2011), intracellular bacterial symbionts of fungi remain largely unexplored.

Bacteria living inside fungal cells were first well documented in the AM fungus *Geosiphon pyriformis*. The symbiosis of this fungus with the filamentous cyanobacteria *Nostoc puntiforme* is the only known example of fungal endocyanosis to date. Interestingly, this intracellular symbiosis is cyclical, which means that the incorporation of free-living cyanobacteria within the fungal cytosol occurs periodically and only when the cyanobacteria are in the appropriate developmental state (Mollenhauer et al., 1996; Wolf and Schüßler, 2005). This cyclical transmission resembles the one that exists between plants and rhizobia, and plants and mycorrhizal fungi. *N. puntiforme* has the capability of establishing symbiosis not only with *Geosiphon*, but also with gymnosperm cycad *Macrozamia* sp. (Rippka and Herdman, 1992). Despite its endosymbiotic nature, the genome size of *N. puntiforme* is quite large (8.9 Mb), where 29% of the predicted genes seemed to be unique to this free-living and symbiotic cyanobacteria (Meeks et al., 2001).

Besides the endocyanosis, *G. pyriformis* harbors other type of intracellular bacteria, which were previously referred to as “bacterial-like organisms.” Recent molecular studies on these bacterial-like organisms have revealed that these endofungal bacteria belong to a monophyletic clade of the *Mollicutes*, which form a sister group to *Mycoplasmatales* and *Entoplasmatales* (Naumann et al., 2010). These obligate and hereditable bacterial symbionts are widespread in several lineages of AM fungi including *Archeosporales*, *Diversisporales* and *Glomerales*, which suggests that this symbiosis preceded the diversification of AM fungi and thus, that it may be as old (about 400 million years) as the symbiosis of AM fungi with plants (Naumann et al., 2010). Currently, no genomic information about these diverse *Mollicutes-*related symbionts is available. Furthermore, and largely due to their unculturability, their characteristics, functional roles and capacities remain cryptic.

Among AM fungi, the best-studied system in terms of their bacterial endosymbionts is the AM fungus *Gigaspora margarita* BEG34. Pioneering studies on this AM fungus clearly showed that it harbored an intrahyphal, gram-negative and vertically transmitted β-proteobacteria from the *Burkholderiaceae* family named “*Candidatus* Glomeribacter gigasporarum” (Bianciotto et al., 1996, 2003, 2004). In this relationship, the fungus is an obligate symbiont of plants, but facultative with respect to its bacterial symbiont (Desirò et al., 2014b). Studies made with cured/endobacterium-free fungal spores indicated that *G. margarita* BEG34 can survive, without its bacterial partner, albeit showing signs of a diminished ecological fitness (Lumini et al., 2007). However, “*Ca*. Glomeribacter gigasporarum,” although it can be extracted from the fungus, it remains unculturable in laboratory settings (Salvioli et al., 2008). It has been shown that in several strains of *G. margarita* both, the gram-positive *Mollicutes*-related bacteria as well as the gram-negative “*Ca*. Glomeribacter gigasporarum,” may be present (Desirò et al., 2014b).

The genome of “*Ca*. Glomeribacter gigasporarum” revealed that these endobacterium possess a reduced genome (1.72 Mbp) with a relatively high G+C content of 54.82%, and a notorious dependency on its host for carbon, phosphorus and nitrogen as well as energy which is likely obtain from transporting and degrading amino acids (Ghignone et al., 2012). Phylogenetically, *“Ca*. Glomeribacter gigasporarum” closest relative is the also endofungal bacteria *B. rhizoxinica*, but analyses based on the metabolic pathways and their completion grouped this bacterium closer to some other endosymbionts of insects like “*Candidatus* Hamiltonella defensa,” *Wolbachia* spp. and *Wigglesworthia glossinidia*, suggesting that both fungal and insect intracellular symbionts had undergone convergent evolution (Ghignone et al., 2012).

A contrasting bacterial–fungal symbiosis was reported on *R. microsporus*, a zygomycete fungus, which was known as the causal agent of rice seedling blight thanks to the production of the toxin and potent antitumor agent rhizoxin (Gho et al., 1978; Takahashi et al., 1987). Partida-Martinez and Hertweck (2005) demonstrated that rhizoxin is not produced by the fungus, but by intracellular living bacteria from the Genus *Burkholderia*. The successful isolation and cultivation of the bacteria without its fungal host provided direct evidence of the bacterial origin of rhizoxin and their derivatives (Partida-Martinez and Hertweck, 2005, 2007; Scherlach et al., 2006), and paved also the way for establishing a model for bacterial-fungal symbioses. All endosymbiotic bacteria associated to toxinogenic *R. microsporus* formed a defined cluster among *Burkholderia* and were later taxonomically defined as *B. rhizoxinica* and *Burkholderia endofungorum* (Partida-Martinez et al., 2007b), the latter being the type strain which besides rhizoxin, is able to produce the ciclopeptide toxin rhizonin A (Partida-Martinez et al., 2007a). Strikingly, it was also demonstrated that cured, that is *R. microsporus* strains in which the bacteria has been eliminated by antibiotic treatment, are unable to form asexual sporangia and sporangiospores, meaning that the fungal host depends on endobacteria not only for the production of “mycotoxins,” but also for its asexual reproduction. These experiments also confirmed that bacteria are transmitted vertically, as single bacterial cells were encapsulated in fungal spores, warranting the maintenance of the symbiosis along generations (Partida-Martinez et al., 2007c).

Further evolutionary studies on the *Rhizopus*–*Burkholderia* symbiosis have suggested that it is not as antique as the one between the AM fungi *G. margarita* and “*Ca.* Glomeribacter gigasporarum” (Castillo and Pawlowska, 2010), and that it likely emerged as a shift from parasitism to mutualism, as mucoromycotina and some branches of the Ascomycota independently evolved rhizoxin resistant mechanisms before the establishment of the symbiosis (Schmitt et al., 2008).

Analyses of the first published endofungal genome of the type strain of *B. rhizoxinica* revealed a relatively reduced genome of 3.75 Mbp. These analyses suggested that *B. rhizoxinica* is in an early phase of endosymbiosis, as many pseudogenes, MGEs and transposons are present in its genome (Lackner et al., 2011a,b). Interestingly, and in contrast to other symbiotic systems which are also predicted to be in an early phase of evolution such as the chromatophore in *Paulinella chromatophora* (Nowack et al., 2008), the genome of *B. rhizoxinica* has shown that around 30.8% of its coding genes had no homology to any other organisms sequenced to date. This means that *B. rhizoxinica*’s genome is not simply a subset of genes derived from free-living *Burkholderia* spp. (Lackner et al., 2011a).

Despite this moderate genome reduction and evidence of a genome in transition, it is striking the great dependency of both partners to each other. These facts prompt questions such as: how fast can intracellular mutualisms between bacteria and fungi be established? Which molecular mechanisms had contributed to this dependency? Under which circumstances would the *Burkholderia* endosymbionts evolve to a completely reduced genome close to the status of an organelle? Are host switching and/or population effective size contributing to genome stability in this symbiosis?

Very recently, the discovery of *Mollicutes*-related endobacteria not only in Glomeromycota, but also inside several strains of *Endogone* (Desirò et al., 2014a), a fungal genus belonging to Mucoromycotina which forms intimate associations with the earliest groups of land plants, has raised further questions about the ecological and evolutionary relevance of endofungal bacterial symbionts in the establishment of the symbiosis of plants with fungi and ultimately, in the establishment in land by plants (Bidartondo et al., 2011). All these reports lend support to the idea that bacterial endosymbiosis in fungi is ancient and may have started within ancestral fugal members characterized by coenocytic mycelium (Desirò et al., 2014a).

Certainly, further comprehensive molecular studies from the aforementioned as well as other bacterial–fungal symbiosis known to date will shed light on the evolutionary patterns of genome reduction and stability in these systems. Recent reports on intracellular gram-negative bacteria producing *N*-homoserine lactones in the zygomycete *Mortierella alpina* A-178 (Kai et al., 2012), together with diverse bacteria associated to endophytic fungi of the Ascomycetes (Hoffman and Arnold, 2010), and the endobacterial communities associated with the ectomycorrhizal fungus *Laccaria bicolor* (Bertaux et al., 2005) are expanding the universe of close interactions between bacteria and fungi, enabling a deeper understanding of the commonalities and differences between these symbioses and the best known bacteria-insect models. Moreover, some genome projects from fungi and bacteria engaged in intracellular symbioses had been recently undertaken as indicated in the website of the Department of Energy Joint Genome Institute (Nordberg et al., 2014). Thus, the consequences of such endosymbioses may be soon evaluated from both sides of the partnership.

## DRIVERS OF GENOME REDUCTION IN HOST-ASSOCIATED BACTERIA

The process of genome reduction in host-associated bacteria is largely determined by the intracellular environment in which they live. Specifically, it is reasoned that genes unnecessary for living in intracellular conditions are not maintained by selection and lost along evolution. The process of genome reduction has been documented and seem to follow common trends from one stage of reduction to the next (Toft and Andersson, 2010). However, the mechanisms that drive these changes are far from established. In this section, we will review some of the more prominent and recent hypothesis about what drives genome reduction in host-associated bacteria.

Currently one of the most prominent and widely accepted hypotheses to explain genome reduction is based on the process known as the Muller’s ratchet, which states that in populations undergoing constant bottlenecks and no recombination, genome reduction occurs through the accumulation of slightly deleterious mutations (Moran, 1996; McCutcheon and Moran, 2012). Under these conditions selection fails to retain genes which then, by the constant accumulation of mutations, become inactive and are eventually deleted from the genome. As a result, several of the typical characteristics of these genomes, like their large A+T content or their small genomes, reflect known mutational biases (i.e., G:C to A:T mutations and deletions over insertions) rather than adaptations evolved by selection (Moran, 2003; Moya et al., 2008; McCutcheon and Moran, 2012).

In agreement with this hypothesis, theoretical studies suggest that proteins in *Buchnera* are less stable as a consequence of accumulating slightly deleterious mutations over large periods of time (van Ham et al., 2003). Additionally, proteomic studies demonstrate that the chaperonin GroEL is one of the most abundant proteins in *Buchnera* (Poliakov et al., 2011). As such, it is believed that GroEL plays a central role by stabilizing an otherwise unstable proteome (Moran, 1996; Fares et al., 2002). Furthermore, it has been shown that GroEL has suffered substitutions due to positive natural selection in two important functional regions of the protein which were suggested to be involved in the optimization of the ability to bind and prevent inappropriate folding of GroEL in *Buchnera* spp. and Flavobacteria endosymbionts (Fares et al., 2005). Also supporting this hypothesis, the pattern of non-synonymous (*d_N_*) versus synonymous (*d_S_*) substitutions (*d_N_*/*d_S_*) among 42 pairs of closely related bacteria is consistent with genetic drift driving the process of genome reduction. Accordingly, *d_N_*/*d_S_* is consistently larger in organisms with smaller genomes (Kuo et al., 2009).

However, other processes have been suggested to explain the evolution of reduced genomes. For instance, Itoh et al. (2002) suggested that the acceleration of molecular evolution experienced in these genomes is due to a general increase in the mutation rate rather than to the Muller’s ratchet mechanism. This hypothesis is based on: (a) the fact that the genomes of obligate mutualistic bacteria often lack DNA repair genes; (b) Muller’s ratchet hypothesis is not consistent with the fact that the genomes of mutualistic endosymbionts, like those of *Buchnera* spp. have 100s of millions of years of existence; and (c) the pattern of acceleration of molecular evolution of *Buchnera* spp. proteins is consistent with increase of mutation rate and not with relaxation of purifying selection. In fact, the authors propose that loss of DNA repair genes is one of the necessary prerequisites to evolve a reduced genome. This hypothesis resembles the one proposed by Marais et al. (2008) which also suggest that the increased mutation rate causes genomic reduction in free-living bacteria. However, lack of repair genes coupled with recurrent bottlenecks and no recombination sets the conditions for evolution by Muller’s ratchet.

Whatever the cause of the acceleration of the rate of evolution is, the hypothesis that these bacteria accumulate slightly deleterious mutations has to explain how these organisms manage to survive despite millions of years of existence. In this sense, compensatory evolution has to be part of the answer which suggest that a mutation may be compensated by a second mutation which returns the system to a working state (Kern and Kondrashov, 2004; Fares et al., 2005; McCutcheon and Moran, 2012). An example of which is the afore mentioned chaperon overexpression which help the organism to tolerate more mutations by lowering the threshold of the free energy necessary to fold properly compensating for the introduction of destabilizing mutations and making the system more robust (Gros and Tenaillon, 2009).

Importantly, the Muller’s ratchet hypothesis, although very compelling in the latter stages of reduction, falls short in the early stages where symbionts lack many of the prerequisites for this process to occur. Such as in facultative pathogens, which due to their ability to return to a free-living state can evade bottlenecks and have larger population sizes. In addition, the early stages of reduction in pathogens are characterized by acquisition of genes by HGT as well as their rapid modification by recombination (Toft and Andersson, 2010).

Traditionally, genome reduction in host-associated bacteria has been linked to this view of evolution based on the relaxed or neutral selection coupled to genetic drift, since it better explains the presence of non-functional DNA such as ancient pseudogenes and intergenic regions commonly found in these bacteria (Mira et al., 2001; Dutta and Paul, 2012; McCutcheon and Moran, 2012). However, a recent shift in this view has started to appear in the form of empirical evidence (Koskiniemi et al., 2012; Lee and Marx, 2012; D’Souza et al., 2014), and new hypothesis which view genome reduction of host-associated bacteria as a selection-based process, at least on its early stages (Mendonça et al., 2011; Bliven and Maurelli, 2012; Morris et al., 2012). Here we will review a few of these hypotheses.

The first is an interesting novel hypothesis which suggests selective gene loss based on the loss of robustness in predictable environment. In this case, robustness is defined as the ability of an organism to withstand harsh and variable environments as well as coping with internal changes and perturbations in the inner workings of the cell. The hypothesis predicts that under predictable environments such as the interior of the cell, this robustness is not required and thus, genomic reduction would be observed. In correlation with this, the authors found empirical evidence of the existence of a selective drive to retain protein family diversity by sacrificing redundancy of functions. Here, redundancy of function, being a form of robustness. In other words, reduced genomes tend to have more protein families but each family tends to have very few members. Thus the authors suggest that the probability of losing a gene is higher if multiple copies of redundant genes exist, but very small if the function is unique. This also indicates that only those paralogs with similar functions will be lost. Finally, they suggest that other forms of robustness such as network redundancy may be similarly affected. For instance, the protein family composed of the transketolases TktA (EC2.2.1.1), TktB (EC 2.2.1.1), and the 1-deoxyxylulose-5-phosphate synthase Dsx (EC2.2.1.7) in *Escherichia coli* provides an example of this. The transketolases are 99% identical to each other but only 29% with respect to Dsx. In *B. aphidicola,* only one transketolase and one 1-deoxyxylulose-5-phosphate synthase remain (Mendonça et al., 2011).

Although originally proposed for free-living organisms, the BQH may also play an important role in genome reduction of host-associated bacteria. A study by D’Souza et al. (2014) showed that 76% of 949 sequenced bacteria were auxotrophic for at least one of 25 different metabolites needed for growth (20 amino acids, 3 vitamins, and 2 nucleosides), of which endosymbiotic bacteria where the most commonly observed auxotrophs (91% of endosymbiotic bacteria where auxotrophs for at least one of the 25 metabolites as opposed to 85% for free-living and 64% gut-inhabiting bacteria). Additionally, they observed that when supplemented with the metabolite, auxotroph strains of *E. coli* and *Acinetobacter baylyi* showed a significant increase in fitness as compared to the wild type. The selective advantage depended on the concentration of the metabolite, the metabolite in question and the absence or presence of a competitor (D’Souza et al., 2014).

And finally, another hypothesis that supports selection as the driver to reduction is the AVG hypothesis. This theory, proposed for pathogens, states that once a pathogen colonizes a new niche, its new role as a pathogen may be hindered by the expression of genes present and required in its previous environment. In order to better adapt and fulfill its role as a pathogen, these AVGs are selected against and end up inactivated or deleted. This theory is based on the concept of antagonistic pleiotropy, which states that the same gene may have adverse fitness in different environments. And thus, the AVG hypothesis may be considered not only for pathogens but also for other forms of symbiosis. An example of an AVG gene is that of *speG* in *Shigella* species. This gene codes for a spermidine acetyltranferase that generates *N*-acetylspermidine from spermidine. The loss of this gene prevents spermidine metabolism and allows for high levels of this compound in the cell. High spermidine concentration is correlated to higher survival to oxidative stress, which is of particular importance for *Shigella* spp. since part of it life cycle includes being swallowed by macrophages, and withstanding severe oxidative stress. Thus, the loss of this gene confers a higher fitness (Bliven and Maurelli, 2012).

## CONCLUSION

In Monterroso (1959) the Latin-American writer Augusto Monterroso wrote one of the smallest stories on Spanish language called “The dinosaur”: “*Cuando despertó, el dinosaurio aún estaba allí.*” An approximate English translation would be “When he awoke, the dinosaur was still.” The story is composed of two parts separated by a coma. In the first one, a tacit subject awakes. In the second one, the subject realizes that a dinosaur, an explicit subject, “was still.” Despite of its small size, all the elements of a story (i.e., character, setting, plot, conflict and theme) are present in these just seven words, although some of these elements are implicit and left to the imagination of the reader.

Similarly, in the case of host-associated bacteria, extreme genome reduction is possible by metabolic and functional integration with the host and with other co-symbionts. And in the case of free-living bacteria, the BQH suggest that selection will favor the loss of those genes that code for expensive functions that are anyway provided as PGs by other species (Morris et al., 2012). In both cases the outcome is dependence between different cellular lineages. And, as in the case of Augusto Monterroso’s story, in which some parts of the story are left implicit to the reader, parts of the functions required by the cells are performed outside their boundaries.

What cellular status do prokaryotes with extremely reduced genomes deserve? Perhaps the symbionelle concept is part of the answer (Reyes-Prieto et al., 2014). The symbionelle concept was constructed to accommodate those cases of endosymbionts that fail to reach a minimal gene set (Gil et al., 2004). They possess evolved genomes with so few genes that they are not able to perform the three basic functions of present day cells without the presence of a host and/or other co-symbionts, and so, represent a new category. These symbionelles present evolutionary convergence with organelles exhibiting clear and important similarities and distinctions, although each evolved in completely different evolutionary scenarios, where organelles evolved before multicellular life and symbionelles distinctly throughout insect evolution. (Reyes-Prieto et al., 2014).

A concluding remark is made by Wolf and Koonin (2013), in which they suggest that genome reduction is the dominant form of evolution in a two phase genomic model where a short phase of abrupt increase in complexity, and thus genomic size, permits innovation while a long phase defined by genomic reduction allows for adaptation. Once again, demonstrating the importance of dependence as a form of adaptation born from symbiotic interactions. And finally, as pointed out by (Sloan and Moran, 2013), the biology of obligate intracellular mutualistic bacteria offers the opportunity to study the evolutionary process acting on different levels of biological organization. Thus, the development of a multilevel theory of causation stands at the frontier of evolutionary theory (Gould, 2002).

## Conflict of Interest Statement

The authors declare that the research was conducted in the absence of any commercial or financial relationships that could be construed as a potential conflict of interest.

## ABBREVIATIONS

AM: arbuscular mycorrhizal
AVG: anti-virulence gene
dN/dS: nonsynonymous versus synonymous substitutions
HGT: horizontal gene transfer
IS: insertion sequence
MGEs: mobile genetic elements
Trp: tryptophan
UGA_stop_: UGA stop-coding codon
UGA_trp_: UGA tryptophan-coding codon.
